# Supplementary material for: Factors Associated with Frequency of Peanut Consumption in Korea: A National Population-Based Study
Source: Nutrients. 2020 Apr 25;12(5):1207. doi: 10.3390/nu12051207 (PMC7282004; doi:10.3390/nu12051207)
Supplement: Supplementary file 1 [file nutrients-12-01207-s001.pdf]

Supplement materials

Factors Associated with Frequency of Peanut Consumption in Korea: A National Population-Based Study

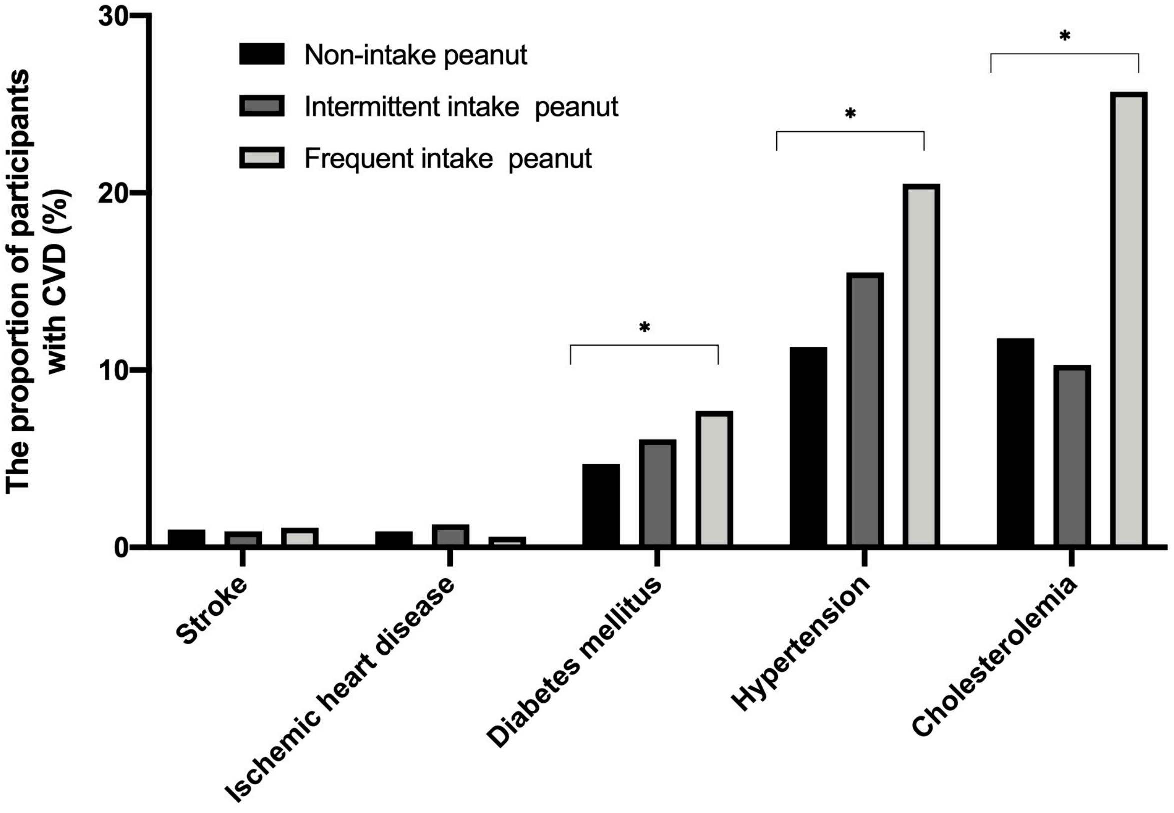

Figure S1. The weighted proportion of cardiovascular diseases and diabetes mellitus by peanut intake groups

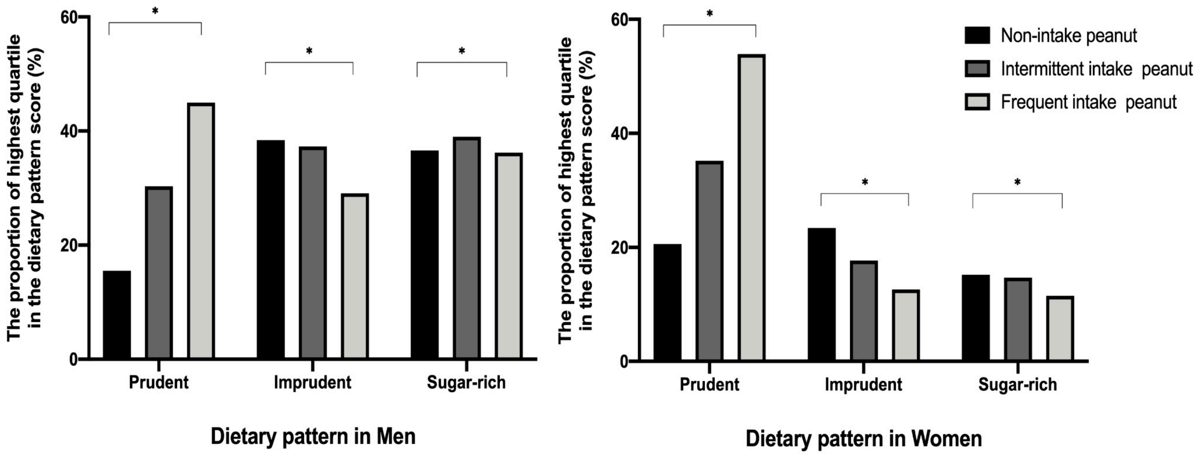

Figure S2. The weighted proportion of the highest quartile of dietary pattern score by peanut intake groups in men (A) and women (B)

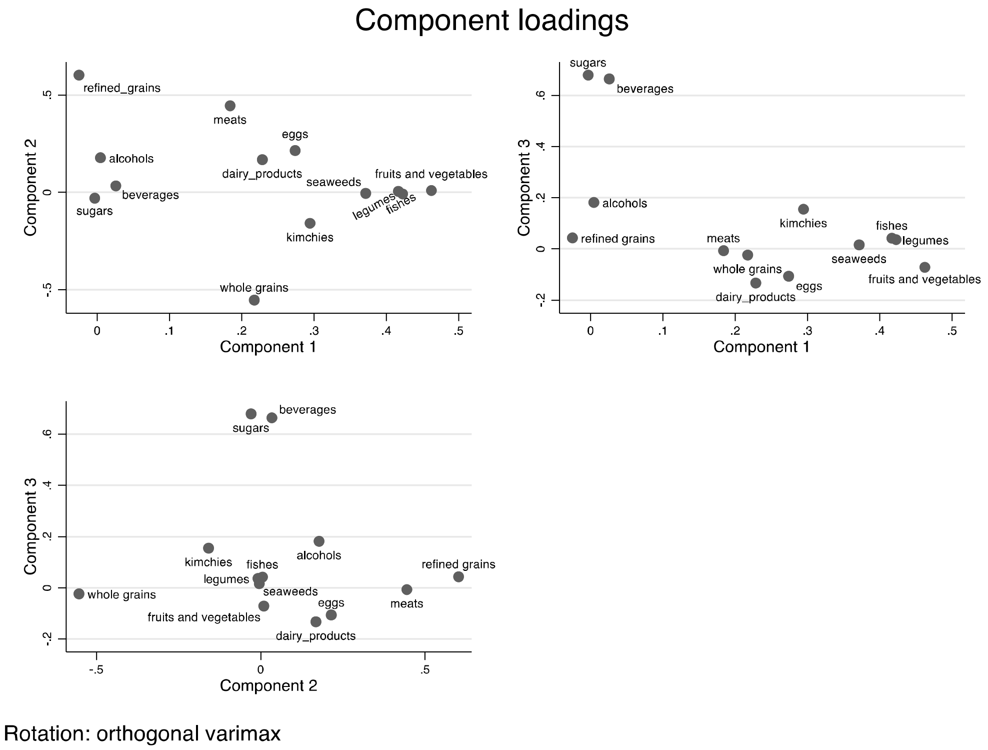

Figure S3. Principal component analysis plots of three principal components.

Table S1. Component foods of the 13 food groups.

**Food Group**	Component food
Red and white meat	*Seolleongtang*, *Budaejjigae*, processed meats, roasted pork belly, pork chop, pork stir-fried, *Tangsuuk*, beef raw, roasted meat, *bulgogi*, ham, *Sundae*, *Samgyetang*, chicken stir fry, fried chicken, duck meat
Dairy products	Whole milk, butter, liquid yogurt, mussel yogurt, ice-cream
Fruits and Vegetables	Bean sprouts, spinach sprouts, bellflower, pumpkin, other herbs, cucumber, radish, vegetable salad, green onion, *ssam* vegetables, boiled broccoli, garlic, lotus root stew, fried mushrooms, potato, steamed potato, steamed sweet potato, steamed corn, strawberry, tomato , melon, watermelon, peach, grape, apple, pear, persimmon, tangerine, banana, orange, kiwi
Refined grains	Rice, *Bibimbap*, *Gimbap*, curry rice, ramen, noodle, *Jjajangmyeon*/*Champon*, cold noodle, rice cake soup, dumplings, bread, sweet red bean bread, castella, pizza, snow white rice cake, *Tteokbokki*, pancake, Japchae, cookies, snacks
Whole grains	Whole grain, cereals
Sugar	Jam, sugar, chocolate, coffee prim
Fishes	Pollack stew, pollack soup, mackerel, hairtail, anchovy, squid, crab, salted shrimp, fish cake
Legume	Miso soup, miso stew, tofu stew, tofu, bean stew, seasoning soy paste, soy milk
Egg	Fried egg, boiled egg
Kimchi	*Kimchi* stew, *Kimchi*, other type of *Kimchi*, pickles
Beverage	Coffee, green tea, soda, fruit juice, parched rice powder shake
Alcohol	Beer, rice wine, *Soju*
Seeweeds	Grilled seaweed, green onion, seaweed

Table S2. Sex-stratified sociodemographic characteristics of study participants according to frequency of peanut consumption.

Variable	Male (n=6,998)				Female (n=10,672)			
	Non-intake (n=3,847)	Intermittent intake (n=3,030)	Frequent intake (n=121)	*P* value	Non-intake (n=6,705)	Intermittent intake (n=3,696)	Frequent intake (n=226)	*P* value
Age group				<0.001				< 0.001
20s	69.8 (1.5)	29.3 (1.5)	0.9 (0.2)		79.0 (1.2)	20.3 (1.2)	0.7 (0.2)	
30s	60.0 (1.4)	39.1 (1.4)	0.9 (0.2)		73.1 (1.0)	26.1 (1.0)	0.7 (0.2)	
40s	52.8 (1.4)	46.4 (1.4)	0.8 (0.2)		60.2 (1.1)	38.5 (1.1)	1.3 (0.2)	
50s	48.1 (1.4)	49.2 (1.4)	2.7 (0.4)		52.2 (1.2)	44.2 (1.2)	3.5 (0.4)	
60s	48.9 (2.0)	47.5 (2.0)	3.6 (6.9)		55.1 (1.7)	39.5 (1.6)	5.3 (0.8)	
Resident area				0.616				0.551
Rural	57.4 (0.8)	41.3 (0.8)	1.3 (0.2)		65.0 (0.6)	33.1 (0.6)	1.9 (0.2)	
Urban	56.6 (1.8)	41.7 (1.7)	1.7 (0.4)		65.2 (1.3)	33.3 (1.3)	14.7 (0.3)	
Household income				<0.001				<0.001
Lowest	65.8 (2.4)	32.9 (2.4)	1.3 (0.5)		70.5 (1.7)	27.6 (1.6)	1.9 (0.4)	
Middle-low	60.3 (1.5)	38.5 (1.5)	1.1 (0.3)		67.6 (1.0)	30.7 (1.0)	1.7 (0.2)	
Middle-high	57.7 (1.2)	41.1 (1.3)	1.2 (0.3)		64.3 (1.0)	34.1 (1.0)	1.6 (0.2)	
Highest	52.8 (1.2)	45.4 (1.2)	1.8 (0.3)		62.1 (1.0)	35.7 (1.0)	2.2 (0.3)	
Household composition				0.912				<0.001
Living alone	57.9 (1.5)	40.8 (1.5)	1.3 (0.3)		62.7 (1.2)	34.8 (1.2)	2.5 (0.3)	

Single generation household	57.1 (0.9)	41.5 (0.9)	1.4 (0.2)		65.3 (0.7)	33.1 (0.7)	1.6 (0.2)	
Multigeneration household	56.9 (2.4)	41.8 (2.4)	1.3 (0.5)		67.9 (1.7)	29.7 (1.7)	2.4 (0.1)	
Education level				<0.001				0.035
<Elementary	61.0 (2.6)	36.6 (2.6)	2.4 (0.1)		63.0 (0.2)	34.1 (1.7)	2.9 (0.6)	
Middle school	60.5 (2.6)	37.8 (2.6)	1.7 (0.6)		59.8 (1.9)	37.8 (1.9)	2.3 (0.5)	
High school	61.0 (1.2)	37.7 (1.2)	1.3 (0.2)		65.1 (0.1)	33.1 (0.9)	1.8 (0.2)	
College or higher	52.8 (1.1)	46.0 (1.1)	1.3 (0.2)		66.8 (0.1)	31.7 (0.9)	1.5 (0.2)	
Occupation				<0.001				0.031
Unemployed	65.6 (1.6)	33.1 (1.6)	1.3 (0.4)		66.0 (0.8)	32.1 (0.8)	1.9 (0.2)	
Unskilled workers	60.0 (2.7)	39.0 (2.74)	1.1 (0.4)		63.8 (1.9)	62.0 (1.9)	1.2 (0.4)	
Non-manual, skilled workers	55.2 (1.0)	43.4 (1.0)	1.3 (0.2)		63.8 (1.0)	34.1 (0.9)	2.0 (0.3)	
Professionals and managers	53.5 (1.7)	44.9 (1.6)	1.6 (0.3)		66.0 (1.4)	32.5 (1.4)	1.5 (0.3)	
Smoking status				<0.001				<0.001
None	61.0 (1.5)	37.7 (1.5)	1.2 (0.3)		63.7 (0.6)	34.4 (0.6)	1.9 (0.2)	
Former	49.2 (1.2)	49.3 (1.2)	1.6 (0.3)		74.4 (2.2)	24.2 (2.2)	1.3 (0.4)	
Current	61.5 (1.1)	37.1 (1.0)	1.4 (0.2)		76.1 (2.0)	22.5 (2.0)	1.3 (0.5)	

Alcohol consumption				0.149				<0.001
None	56.1 (2.3)	41.7 (2.3)	2.1 (0.6)		66.4 (1.3)	31.4 (1.3)	2.1 (0.4)	0.019
Moderate	56.6 (0.9)	42.1 (0.9)	1.3 (0.2)		68.3 (1.0)	29.8 (1.0)	1.9 (0.3)	
Heavy	56.5 (2.6)	41.6 (2.5)	1.8 (0.6)		79.4 (3.5)	18.8 (3.5)	1.8 (0.9)	
BMI				0.038				<0.001
Underweight (<18.5)	65.2 (4.4)	31.3 (4.3)	3.5 (2.0)		73.9 (2.0)	33.8 (2.0)	0.8 (0.3)	
Normal (18.5-24.9)	57.9 (0.9)	40.8 (0.9)	1.3 (0.2)		64.3 (0.7)	33.8 (0.7)	2.0 (0.2)	
Overweight (25.0-29.9)	55.1 (1.3)	43.4 (1.2)	1.4 (0.3)		63.1 (1.2)	35.0 (1.2)	1.8 (0.3)	
Obese (≥30.0)	60.8 (2.9)	38.1 (2.9)	1.1 (0.5)		72.3 (23)	26.0 (2.3)	1.6 (0.6)	
History of cardiovascular disease† or diabetes mellitus	17.4 (0.7)	21.8 (1.0)	24.7 (4.7)	0.005	15.0 (0.6)	19.1 (0.8)	29.6 (3.8)	<0.001

Note: All values are presented weighted percentage and standard error; Study subjects who rarely intake peanut, who intake 0.25 to 4 times per week and who intake 0.7 to 3 times per day were classified into non-intake group, intermittent intake group and frequent intake group, respectively;*Chi-square test was used to estimate the differences sociodemographic factors by peanut intake groups; † Cardiovascular disease was defined as at least one of hypertension, diabetes mellitus, ischemic heart disease, hypercholesterolemia or stroke.; $P < 0.05$ is considered significant.

Table S3. Sex-stratified dietary characteristics of study population according to frequency of peanut consumption.

Variable	Men (n=6,998)				Women (n=10,672)			
	Non-intake (n=3,847)	Intermittent intake (n=3,030)	Frequent intake (n=121)	*P* value	Non-intake (n=6,705)	Intermittent intake (n=3,696)	Frequent intake (n=226)	*P* value
Total energy, kcal/d	2324.98 (15.94)	2566.81 (18.78)	2803.24 (95.7)	<0.001	1791.07 (11.03)	1937.98 (13.93)	1993.73 (54.25)	<0.001
Carbohydrate, g/day	351.72 (2.17)	383.16 (2.51)	407.15 (13.14)	<0.001	287.14 (1.58)	314.17 (2.11)	315.73 (8.86)	<0.001
Protein, g/day	73.53 (0.63)	84.69 (0.81)	95.36 (3.84)	<0.001	60.57 (0.46)	67.13 (0.59)	69.27 (2.13)	<0.001
Total fat, g/day	46.24 (0.51)	53.48 (0.64)	67.33 (3.29)	<0.001	37.76 (0.37)	41.24 (0.47)	47.12 (1.68)	<0.001
Polyunsaturated fatty acid, g/day	11.51 (0.12)	13.96 (0.16)	18.50 (0.86)	<0.001	9.93 (0.09)	11.42 (0.12)	13.87 (0.50)	<0.001
Monounsaturated fatty acid, g/day	14.57 (0.17)	16.91 (0.21)	22.85 (1.11)	<0.001	11.51 (0.13)	12.53 (0.15)	15.37 (0.55)	<0.001
Saturated fatty acid, g/day	14.21 (0.16)	15.67 (0.19)	18.31 (1.01)	<0.001	11.22 (0.12)	11.68 (0.15)	12.18 (0.49)	<0.001
Cholesterol, mg/day	282.3 (3.5)	318.9 (4.4)	350.9 (26.3)	0.007	244.3 (2.6)	260.1 (3.4)	247.6 (13.1)	0.439
Fiber, g/day	19.45 (0.17)	24.28 (0.22)	29.02 (1.19)	<0.001	18.77 (0.14)	23.38 (0.18)	27.41 (0.89)	<0.001
Iron intake, g/day	14.16 (0.11)	16.92 (0.15)	18.98 (0.68)	<0.001	12.49 (0.09)	14.69 (0.11)	15.44 (0.48)	<0.001
Vitamin A RAE, μg/day	600.9 (5.8)	730.6 (7.9)	874.0 (40.6)	<0.001	588.7 (5.2)	696.1 (6.4)	787.2 (31.4)	<0.001
Vitamin B1, mg/day	2.0 (0.2)	2.3 (0.2)	2.5 (0.1)	<0.001	1.7 (0.1)	1.9 (0.1)	2.0 (0.6)	0.792
Vitamin B2, mg/day	1.5 (0.1)	1.6 (0.2)	1.8 (0.1)	0.001	1.3 (0.1)	1.4 (0.1)	1.4 (0.5)	0.238
Vitamin B3, mg/day	14.4 (0.1)	17.3 (0.2)	22.3 (0.8)	<0.001	12.1 (0.1)	14.0 (0.1)	17.0 (0.5)	0.095
Vitamin C, mg/day	93.1 (1.4)	120.9 (1.7)	149.8 (9.3)	<0.001	111.2 (1.3)	144.4 (1.9)	196.9 (10.0)	<0.001

All values are presented weighted mean and standard error; Study subjects who rarely consumed peanut, who consumed 0.25 to 4 times per week and who consumed 0.7 to 3 times per day were classified into non-intake, intermittent intake and frequent intake group, respectively.*ANOVA with Bonferroni correction was used to compare difference between three peanut intake groups

Table S4. Factors associated with frequent peanut consumption in Korean stratified by Sex.

**Variable**	**Male**			**Female**		
	Model 1	Model 2	Model 3	Model 1	Model 2	Model3
**Age**		1.02 (1.01-1.03)*	1.03 (1.02-1.04)*		1.04 (1.03-1.04)*	1.04 (1.03-1.05)*
**BMI**						
Underweight (<18.5)	reference	reference	reference	reference	reference	reference
Normal (18.5-24.9)	1.26 (1.04-1.53)*	1.16 (0.72-1.87)	0.93 (0.52-1.67)	1.13 (0.90-1.40)	1.15 (0.90-1.46)	1.20 (0.80-1.79)
Overweight (25.0-29.9)	1.33 (1.09-1.61)*	1.32 (0.81-2.14)	1.01 (0.56-1.84)	0.98 (0.77-1.25)	0.99 (0.76-1.30)	1.29 (0.84-2.0)
Obese (≥30.0)	1.07 (0.83-1.38)	1.21 (0.71-2.07)	0.99 (0.51-1.91)	0.68 (0.50-0.93)*	0.66 (0.47-0.93)*	0.85 (0.49-1.48)
**Education**						
≤elementary school	reference	reference	reference	reference	reference	reference
Middle school	1.18 (0.86-1.62)	1.20 (0.86-1.67)	1.14 (0.78-1.67)	1.35 (1.09-1.68)*	1.36 (1.08-1.71)*	1.50 (1.03-2.17)*
High school	1.81 (1.41-2.33)*	1.72 (1.32-2.25)*	1.70 (1.23-2.34)*	1.94 (1.62-2.33)*	1.95 (1.59-2.38)*	1.83 (1.32-2.54)*
≥ college	2.34 (1.84-2.99)*	2.22 (1.71-2.89)*	2.22 (1.60-3.11)*	2.12 (1.75-2.57)*	2.04 (1.64-2.53)*	1.90 (1.32-2.74)*
**House income**						
lowest	reference	reference	reference	reference	reference	reference
Middle-low	1.28 (0.99-1.67)	1.29 (0.97-1.72)	1.12 (0.80-1.57)	1.35 (1.10-1.65)*	1.38 (1.11-1.72)*	1.27 (0.92-1.75)
Middle-high	1.43 (1.11-1.82)*	1.45 (1.11-1.88)*	1.17 (0.85-1.61)	1.63 (1.34-1.98)*	1.66 (1.35-2.05)*	1.22 (0.88-1.70)
Highest	1.72 (1.35-2.19)*	1.68 (1.30-2.18)*	1.16 (0.85-1.60)	1.76 (1.45-2.13)*	1.80 (1.46-2.22)*	1.04 (0.74-1.46)
Household composition						

Living alone	reference	reference	reference	reference	reference	reference
Single generation household	1.14 (0.99-1.31)	1.11 (0.95-1.29))	0.99 (0.83-1.19)	1.12 (0.99-1.26)	1.09 (0.96-1.25)	0.84 (0.67-1.04)
Multigeneration household	1.14 (0.91-1.44)	1.08 (0.84-1.39)	0.99 (0.73-1.34)	0.95 (0.79-1.15)	0.97 (0.79-1.18)	0.85 (0.61-1.19)
**Occupation**						
Unemployed	reference	reference	reference	reference	reference	reference
Unskilled workers	1.42 (1.16-1.72)	1.0 (0.76-1.32)	1.06 (0.77-1.47)	0.83 (0.69-0.99)*	0.79 (0.65-0.96)*	0.94 (0.70-1.27)
Non-manual, skilled workers	1.26 (1.07-1.48)*	1.20 (1.01-1.43)*	1.09 (0.87-1.36)	1.09 (0.98-1.21)	1.10 (0.98-1.24)	1.23 (1.02-1.48)*
Professionals and managers	1.42 (1.17-1.72)*	1.29 (1.05-1.60)*	0.85 (0.65-1.11)	1.24 (1.07-1.44)*	1.24 (1.05-1.46)*	1.18 (0.90-1.55)
**History of cardiovascular disease† or diabetes mellitus**	1.0 (0.85-1.17)	0.98 (0.84-1.16)	1.04 (0.85-1.27)	0.84 (0.72-0.98)*	0.89 (0.76-1.04)	0.95 (0.73-1.24)
**Alcohol consumption**						
None	reference	reference	reference	reference	reference	reference
Moderate	1.16 (0.95-1.43)	1.14 (0.92-1.42)	1.06 (0.89-1.34)	1.19 (1.01-1.38)*	1.28 (1.08-1.51)*	1.43 (1.20-1.71)*
Heavy	1.01 (0.76-1.34)	1.05 (0.77-1.44)	1.00 (0.71-1.39)	0.69 (0.43-1.10)	0.87 (0.52-1.46)	1.04 (0.58-1.85)
**Smoking status**						
None	reference	reference	reference	reference	reference	reference
Former	1.26 (1.08-1.47)*	1.23 (1.04-1.46)*	1.09 (0.89-1.34)	0.74 (0.58-0.93)*	0.72 (0.55-0.94)*	0.80 (0.56-1.13)
Current	0.87 (0.74-1.00)	0.81 (0.69-0.96)*	0.78 (0.63-0.96)*	0.64 (0.52-0.81)*	0.66 (0.52-0.84)*	0.67 (0.49-0.93)*
**Prudent dietary pattern**						
1st quartile	reference	reference	reference	reference	reference	reference
2nd quartile	1.66 (1.42-1.95)*	1.70 (1.43-2.02)*	1.69 (1.38-2.06)*	1.81 (1.54-2.12)*	1.82 (1.53-2.15)*	1.83 (1.43-2.34)*

3rd quartile	2.70 (2.31-3.14)*	2.71 (2.29-3.20)*	2.57 (2.11-3.12)*	2.59 (2.25-2.99)*	2.60 (2.22-3.04)*	2.53 (1.94-3.30)*
4th quartile	4.01 (3.39-4.75)*	3.99 (3.31-4.82)	3.82 (3.06-4.76)*	3.71 (3.21-4.27)*	3.78 (3.24-4.41)*	3.80 (2.94-4.91)*
**Imprudent dietary pattern**						
1st quartile	reference	reference	reference	reference	reference	reference
2nd quartile	1.20 (0.99-1.46)	1.16 (0.94-1.43)	0.98 (0.76-1.27)	1.24 (1.09-1.42)*	1.20 (1.05-1.39)*	1.04 (0.82-1.32)
3rd quartile	1.18 (0.98-1.42)	1.18 (0.97-1.43)	1.17 (0.91-1.49)	1.03 (0.89-1.19)	1.01 (0.87-1.18)	0.91 (0.70-1.19)
4th quartile	1.35 (1.11-1.63)*	1.34 (1.10-1.65)*	1.10 (0.85-1.43)	1.08 (0.92-1.26)	1.06 (0.89-1.26)	0.92 (0.69-1.24)
**Sugar-rich dietary pattern**						
1st quartile	reference	reference	reference	reference	reference	reference
2nd quartile	1.21 (1.01-1.45)*	1.20 (0.98-1.46)	1.06 (0.82-1.37)	0.92 (0.81-1.05)	0.96 (0.84-1.10)	1.0 (0.78-1.26)
3rd quartile	1.38 (1.15-1.65)*	1.33 (1.09-1.62)	1.12 (0.86-1.45)	1.01 (0.89-1.14)	1.02 (0.90-1.17)	1.13 (0.90-1.41)
4th quartile	1.15 (0.97-1.37)	1.10 (0.91-1.34)	0.91 (0.70-1.19)	0.84 (0.72-0.97)*	0.88 (0.75-1.04)	0.83 (0.64-1.07)

All values are presented as adjusted Odds Ratio (95% confidence interval). Study subjects who rarely consumed peanuts, who consumed peanuts 0.25 to 4 times per week and who consumed peanuts 0.7 to 3 times per day were classified into non-intake group, intermittent intake group and frequent intake group, respectively.; Model 1 was adjusted for age (years; continuous) and gender.; Model 2 was further adjusted for body mass index (BMI, categorical) and the presence of cardiovascular disease or diabetes mellitus.; Model 3 was further adjusted for levels of education (categorical), household income (categorical), occupation(categorical), current smoking(categorical), alcohol consumption(categorical), and dietary patterns (categorical).† Cardiovascular diseases was defined as at least one of hypertension, diabetes mellitus, ischemic heart disease, hypercholesterolemia or stroke. * P value < 0.05

Table S5. Factors associated with frequent peanut consumption in Korean stratified by history of cardiovascular disease or diabetes mellitus.

Variable	History of CVD or DM (n=3,013)			No history of CVD or DM (n=11,008)		
	Model 1	Model 2	Model 3	Model 1	Model 2	Model3
**Age**	1.02 (1.01-1.03)*	1.02 (1.01-1.03)*	1.03 (1.01-1.05)*	1.03 (1.03-1.03)*	1.03 (1.03-1.03)*	1.03 (1.02-1.04)*
**Sex**						
**Male**	reference	reference	reference	reference	reference	reference
**Female**	0.72 (0.61-0.86)*	0.73 (0.62-0.87)*	0.58 (0.40-0.85)*	0.70 (0.64-0.76)*	0.70 (0.64-0.76)*	0.59 (0.50-0.70)
**BMI**						
Underweight (<18.5)	reference	reference	reference	reference	reference	reference
Normal (18.5-24.9)	1.76 (0.44-6.99)	1.76 (0.44-6.99)	0.36 (0.02-6.04)	1.15 (0.92-1.43)	1.15 (0.92-1.43)	1.16 (0.85-1.60)
Overweight (25.0-29.9)	1.77 (0.44-7.06)	1.77 (0.44-7.06)	0.41 (0.03-6.90)	1.17 (0.94-1.47)	1.17 (0.94-1.47)	1.26 (0.90-1.76)
Obese (≥30.0)	1.48 (0.35-6.20)	1.48 (0.35-6.20)	0.44 (0.02-8.14)	0.93 (0.68-1.27)	0.93 (0.68-1.27)	1.02 (0.66-1.57)
**Education**						
≤elementary school	reference	reference	reference	reference	reference	reference
Middle school	1.40 (1.06-1.85)*	1.39 (1.05-1.84)*	1.07 (0.71-1.66)	1.20 (0.93-1.55)	1.21 (0.93-1.56)	1.39 (0.97-1.99)
High school	1.93 (1.51-2.47)*	1.92 (1.50-2.46)*	1.89 (1.31-2.72)*	1.76 (1.42-2.19)*	1.76 (1.41-2.18)*	1.68 (1.24-2.27)*
≥ college	2.16 (1.60-2.90)*	2.14 (1.59-2.89)*	2.05 (1.33-3.16)*	2.05 (1.64-2.55)*	2.04 (1.64-2.55)*	2.04 (1.49-2.78)*
**House income**						
lowest	reference	reference	reference	reference	reference	reference
Middle-low	1.51 (1.12-2.04)*	1.50 (1.11-2.03)*	1.22 (0.78-1.89)	1.26 (1.00-1.58)*	1.26 (1.00-1.58)*	1.16 (0.86-1.56)

Middle-high	1.80 (1.35-2.40)*	1.78 (1.34-2.37)*	1.01 (0.66-1.57)	1.45 (1.17-1.81)*	1.45 (1.16-1.80)*	1.21 (0.90-1.63)
Highest	2.01 (1.51-2.66)*	1.99 (1.50-2.66)*	1.05 (0.67-1.63)	1.66 (1.33-2.06)*	1.64 (1.32-2.04)*	1.14 (0.85-1.53)
Household composition						
Living alone	reference	reference	reference	reference	reference	reference
Single generation household	1.04 (0.85-1.27)	1.04 (0.85-1.28)	0.87 (0.66-1.15)	1.11 (0.97-1.25)	1.10 (0.97-1.24)	
Multigeneration household	1.11 (0.81-1.52)	1.12 (0.82-1.53)	0.96 (0.60-1.52)	0.99 (0.82-1.19)	0.99 (0.82-1.19)	
**Occupation**						
Unemployed	reference	reference	reference	reference	reference	reference
Unskilled workers	0.80 (0.60-1.06)	0.99 (0.71-1.39)	0.78 (0.51-1.20)	0.89 (0.73-1.08)	0.89 (0.73-1.08)	1.14 (0.88-1.47)
Non-manual, skilled workers	0.95 (0.77-1.16)	0.94 (0.76-1.16)	0.85 (0.61-1.19)	1.15 (1.03-1.29)*	1.15 (1.02-1.28)*	1.24 (1.06-1.45)*
Professionals and managers	0.99 (0.71-1.39)	0.99 (0.71-1.39)	0.74 (0.45-1.21)	1.26 (1.09-1.45)*	1.25 (1.08-1.45)*	1.01 (0.82-1.25)
**Alcohol consumption**						
None	reference	reference	reference	reference	reference	reference
Moderate	1.68 (1.26-2.23)*	1.67 (1.26-2.23)*	1.88 (1.37-2.57)*	1.10 (0.94-1.29)	1.09 (0.93-1.27)	1.12 (0.94-1.33)
Heavy	1.35 (0.85-2.14)	1.34 (0.85-2.12)	1.51 (0.92-2.47)	0.99 (0.74-1.32)	0.97 (0.72-1.31)	1.02 (0.75-1.40)
**Smoking status**						
None	reference	reference	reference	reference	reference	reference
Former	0.95 (0.72-1.26)	0.96 (0.72-1.27)	0.85 (0.60-1.22)	1.01 (0.86-1.18)	1.01 (0.86-1.18)	1.01 (0.83-1.23)
Current	0.68 (0.51-0.92)*	0.69 (0.52-0.92)*	0.69 (0.47-0.99)*	0.72 (0.62-0.83)*	0.72 (0.62-0.83)*	0.73 (0.60-0.89)*
**Prudent dietary pattern**						
1st quartile	reference	reference	reference	reference	reference	reference

2nd quartile	1.73 (1.33-2.24)*	1.73 (1.33-2.24)*	2.00 (1.39-2.88)*	1.77 (1.54-2.03)*	1.76 (1.53-2.02)*	1.67 (1.41-1.97)*
3rd quartile	2.87 (2.22-3.70)*	2.88 (2.23-3.71)*	1.94 (2.07-4.19)*	2.63 (2.30-3.00)*	2.62 (2.30-2.99)*	2.47 (2.07-2.95)*
4th quartile	4.25 (3.28-5.51)*	4.24 (3.27-5.50)*	4.26 (2.96-6.14)*	3.85 (3.34-4.43)*	3.83 (3.33-4.41)*	3.67 (3.06-4.42)*
**Imprudent dietary pattern**						
1st quartile	reference	reference	reference	reference	reference	reference
2nd quartile	1.09 (0.88-1.36)	1.10 (0.88-1.37)	0.86 (0.62-1.18)	1.17 (1.02-1.34)*	1.17 (1.02-1.34)*	1.03 (01.84-1.27)
3rd quartile	1.00 (0.79-1.26)	1.00 (0.79-1.27)	0.97 (0.69-1.39)	1.07 (0.93-1.24)	1.07 (0.93-1.24)	1.09 (0.88-1.35)
4th quartile	1.29 (0.97-1.71)	1.29 (0.97-1.70)	1.12 (0.77-1.64)	1.19 (1.02-1.38)*	1.18 (1.02-1.37)*	1.02 (0.82-1.28)
**Sugar-rich dietary pattern**						
1st quartile	reference	reference	reference	reference	reference	reference
2nd quartile	1.08 (0.83-1.40)	1.07 (0.82-1.38)	1.09 (0.72-1.67)	1.02 (0.89-1.16)	1.03 (0.90-1.17)	1.02 (0.84-1.24)
3rd quartile	1.02 (0.79-1.31)	1.00 (0.78-1.28)	0.89 (0.59-1.33)	1.15 (1.01-1.30)*	1.15 (1.01-1.30)*	1.17 (0.97-1.42)
4th quartile	0.97 (0.75-1.26)	0.96 (0.74-1.24)	0.84 (0.56-1.27)	0.93 (0.91-1.07)	0.93 (0.91-1.07)	0.88 (0.72-1.09)

All values are presented as adjusted Odds Ratio (95% confidence interval). CVD, cardiovascular diseases; DM, diabetes mellitus. Study subjects who rarely consumed peanuts, who consumed peanuts 0.25 to 4 times per week and who consumed peanuts 0.7 to 3 times per day were classified into non-intake group, intermittent intake group and frequent intake group, respectively.; Model 1 was adjusted for age (years; continuous) and sex.; Model 2 was further adjusted for body mass index (BMI, categorical).; Model 3 was further adjusted for levels of education (categorical), household income (categorical), occupation(categorical), current smoking(categorical), alcohol consumption(categorical), and dietary patterns (categorical).† Cardiovascular disease was defined as at least one of hypertension, diabetes mellitus, ischemic heart disease, hypercholesterolemia or stroke. * *P* value < 0.05
